# Supplementary material for: “One-Pot” CuCl2-Mediated Condensation/C–S Bond Coupling Reactions to Synthesize Dibenzothiazepines by Bi-Functional-Reagent N, N′-Dimethylethane-1,2-Diamine
Source: Molecules. 2022 Oct 31;27(21):7392. doi: 10.3390/molecules27217392 (PMC9656399; doi:10.3390/molecules27217392)
Supplement: Supplementary file 1 [file molecules-27-07392-s001.zip › molecules-1950531-supplementary.pdf]

# “One-pot” $CuCl_2$-mediated Condensation/C-S Bond Coupling Reactions to Synthesize Dibenzothiazepines by Bi-functional-reagent N, N’-dimethylethane-1,2-diamine

**Dehe Wang [1†], Qichao Lu [1†], Zhanjun Li [1], Chen Fang [3], Ran Liu [1], Bingchuan Yang [1,4,5*] and Guodong Shen [1,2*]**

[1] School of Chemistry and Chemical Engineering, Liaocheng University, 1 Hunan Avenue, Liaocheng 252000, Shandong, P. R. China
[2] School of Chemistry and Chemical Engineering, Jinan University, 106 Jiwei Road, Jinan 250022, Shandong, P. R. China
[3] Zhejiang Heze Pharmaceutical Technology, 1 Street, Qiantang New District, Hangzhou, 310000, Zhejiang，P. R. China
[4] School of Chemistry and Chemical Engineering, Shandong University, Jinan 250100, P. R. China
[5] The Department of Chemistry, University of South Florida, 4202 East Fowler Avenue, Tampa, Florida 33620, United States

*****Correspondence: shenguodong33@163.com; yangbingchuan@lcu.edu.cn; Tel.:86-635-8239566;

[†]These authors contributed equally to this work.

# List of Content

**1. Copies of NMR spectra of products .................................................................1-17**

1. **Copies of NMR spectra of products**

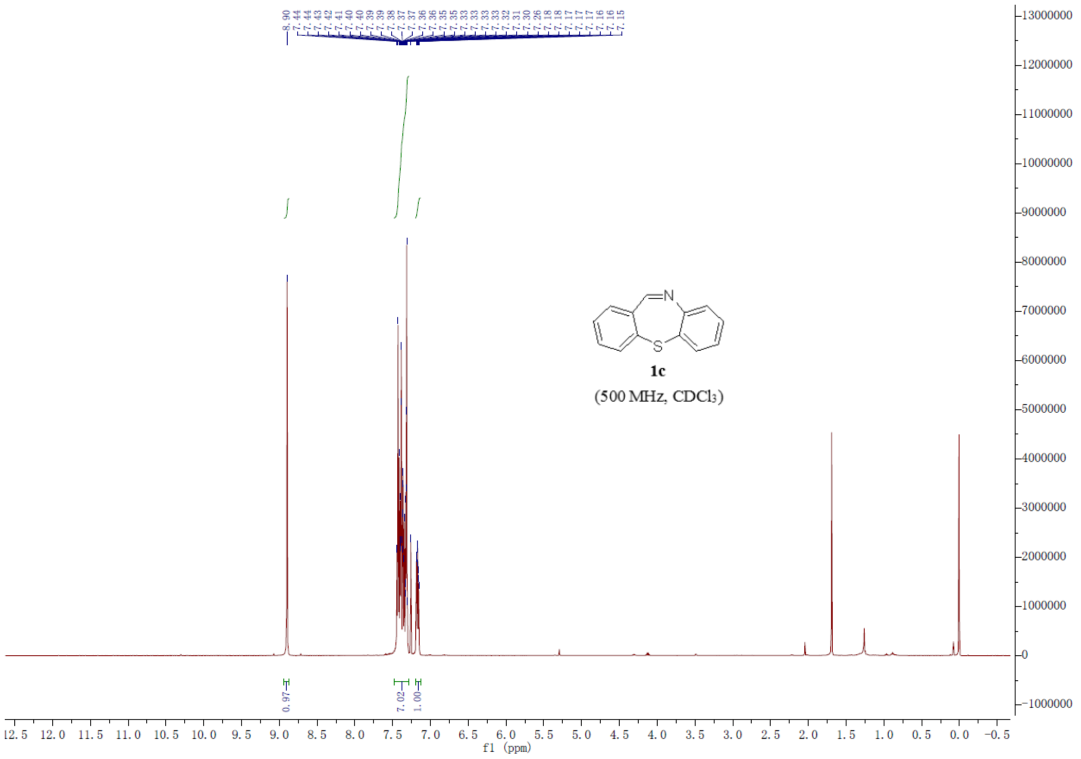

Figure S1: $^1$H NMR ($CDCl_3$) spectrum of Compound (**1c**)

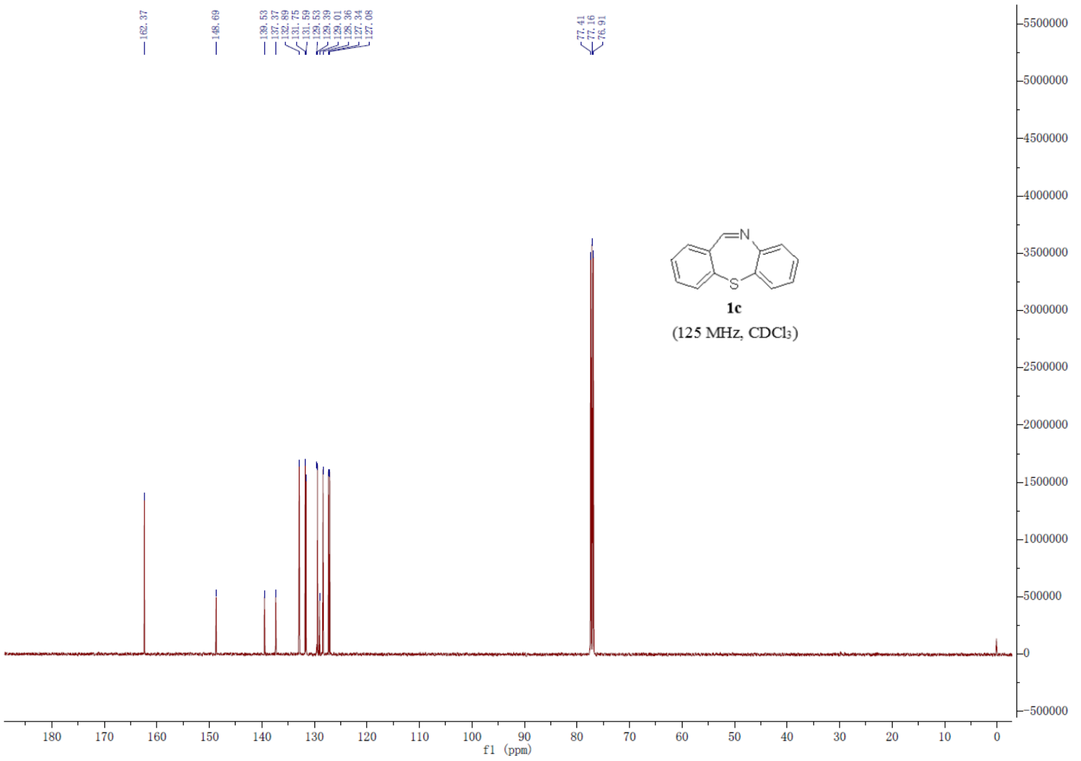

Figure S2: $^{13}$C NMR ($CDCl_3$) spectrum of Compound (**1c**)

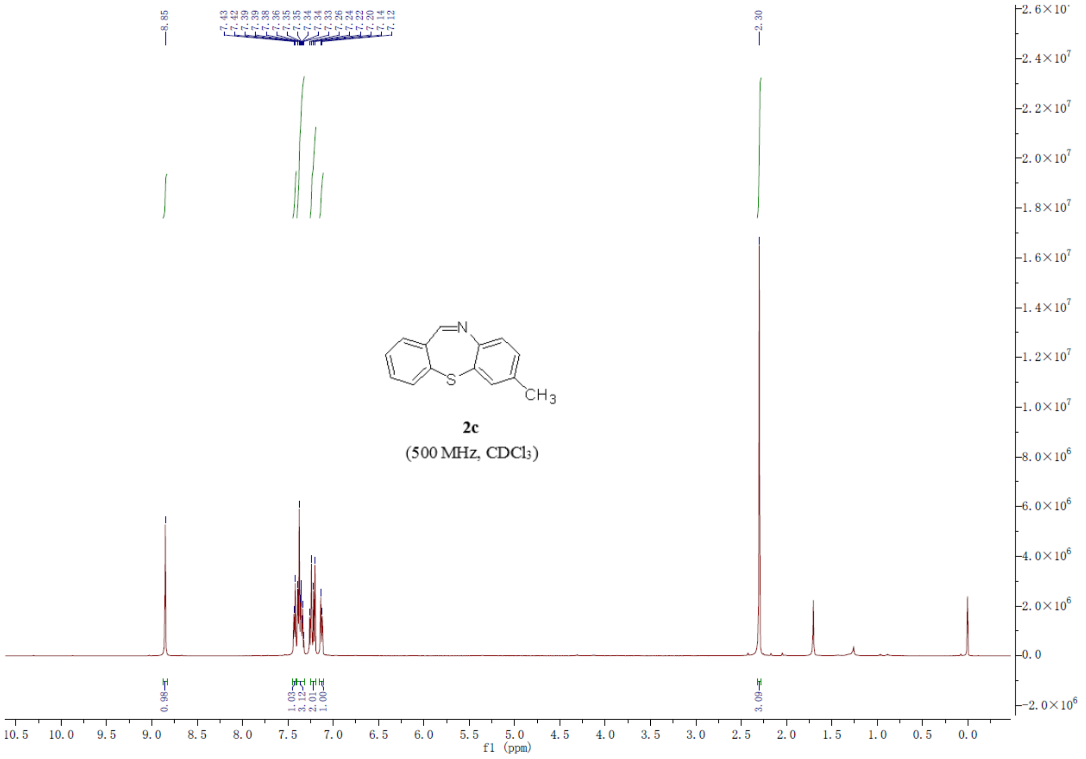


Figure S3: $^{1}$H NMR ($CDCl_3$) spectrum of Compound (**2c**)

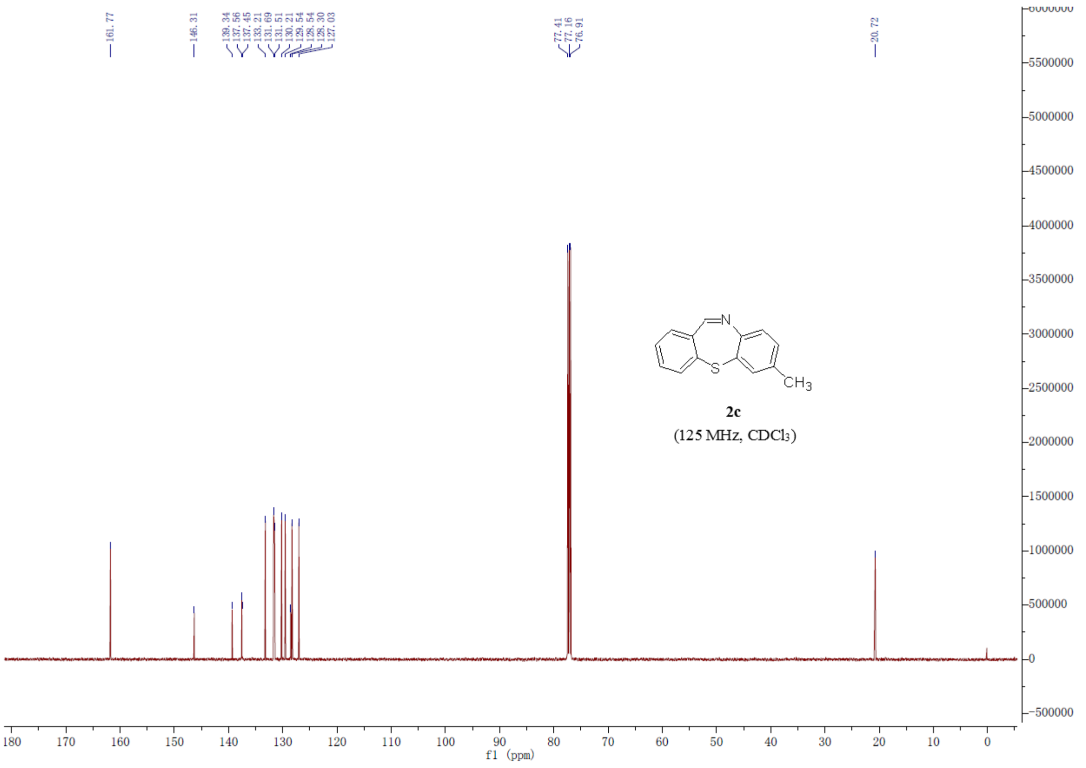


Figure S4: $^{13}$C NMR ($CDCl_3$) spectrum of Compound (2**c**)

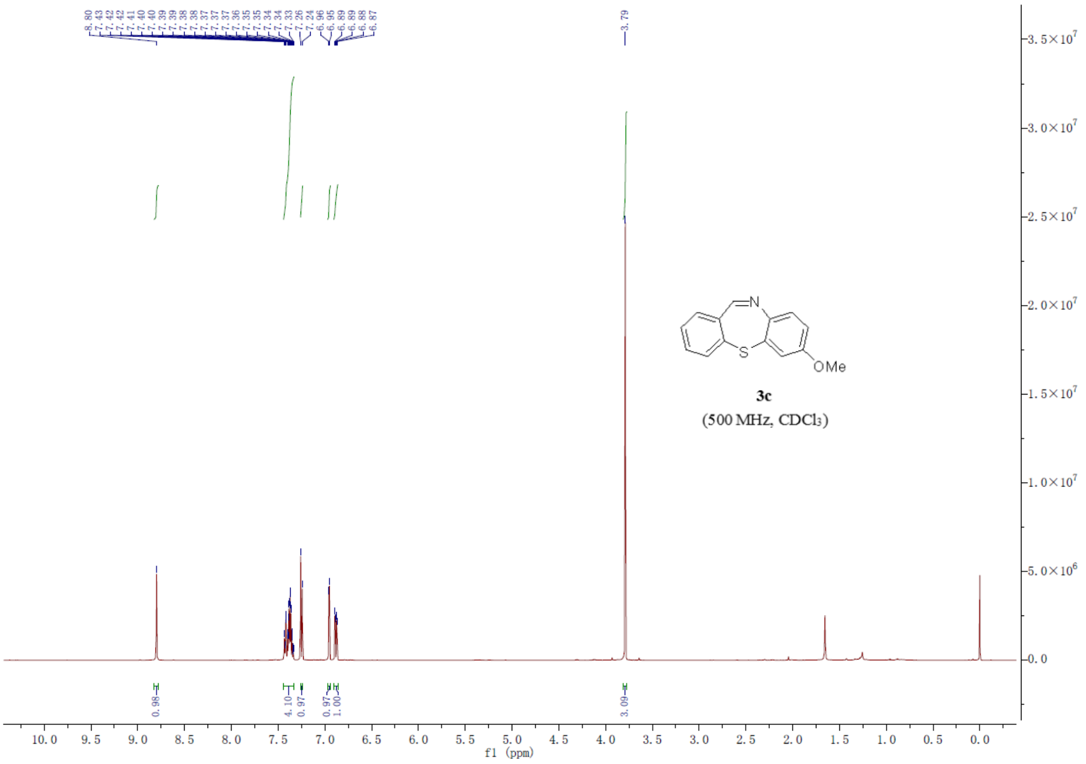


Figure S5: $^{1}$H NMR ($CDCl_3$) spectrum of Compound (**3c**)

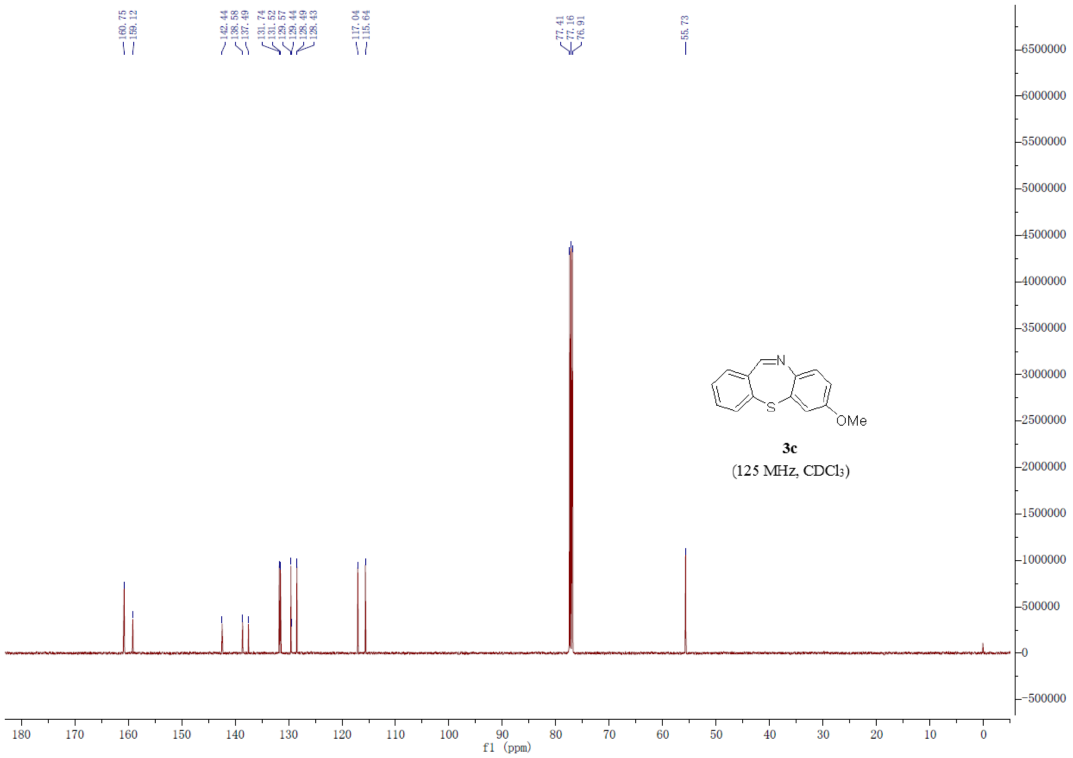


Figure S6: $^{13}$C NMR ($CDCl_3$) spectrum of Compound (**3c**)

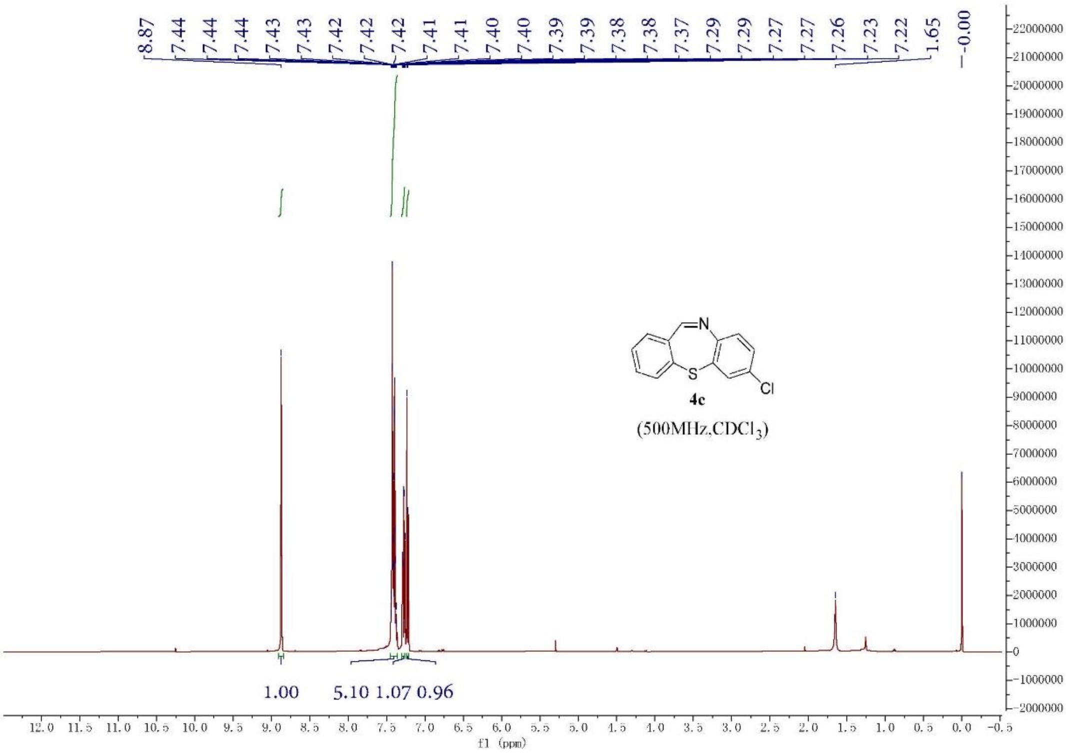


Figure S7: $^{1}$H NMR ($CDCl_3$) spectrum of Compound (**4c**)

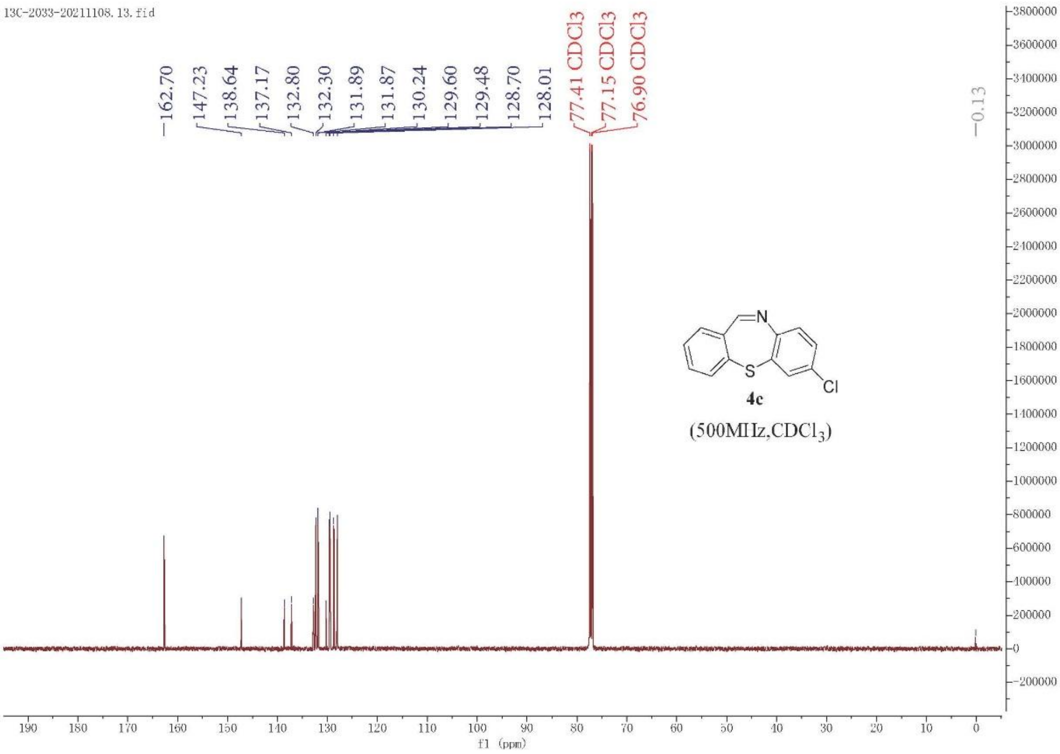


Figure S8: $^{13}$C NMR ($CDCl_3$) spectrum of Compound (**4c**)

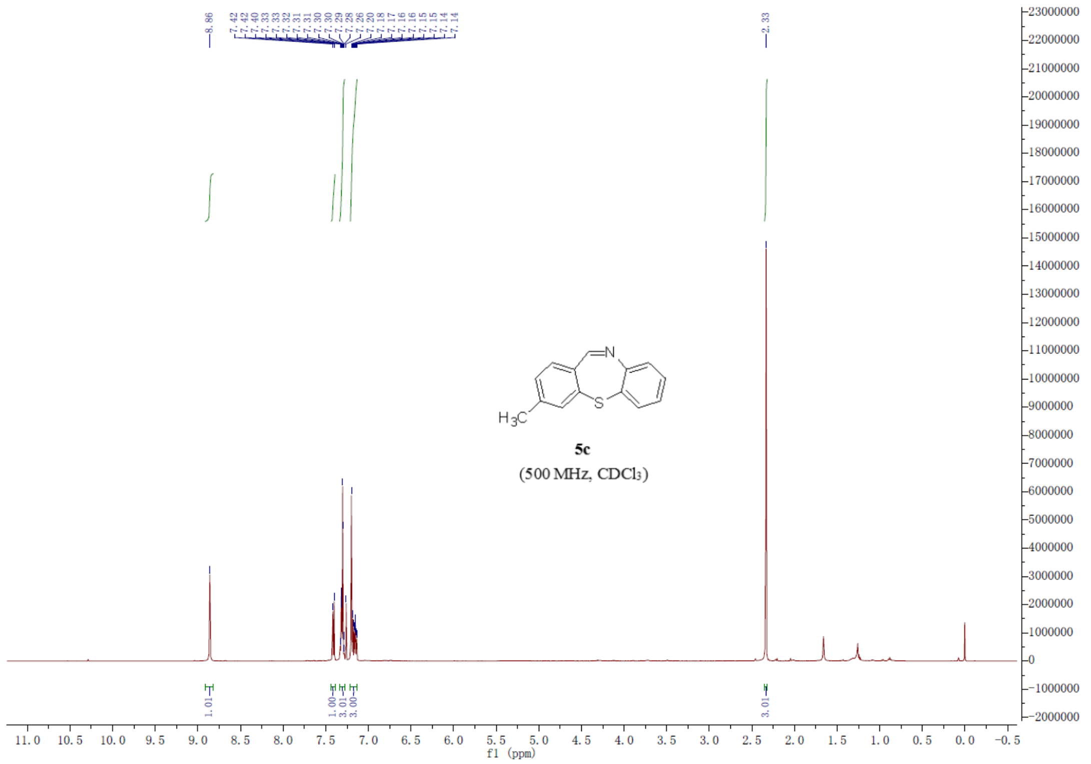

Figure S9: $^{1}$H NMR ($CDCl_3$) spectrum of Compound (**5c**)

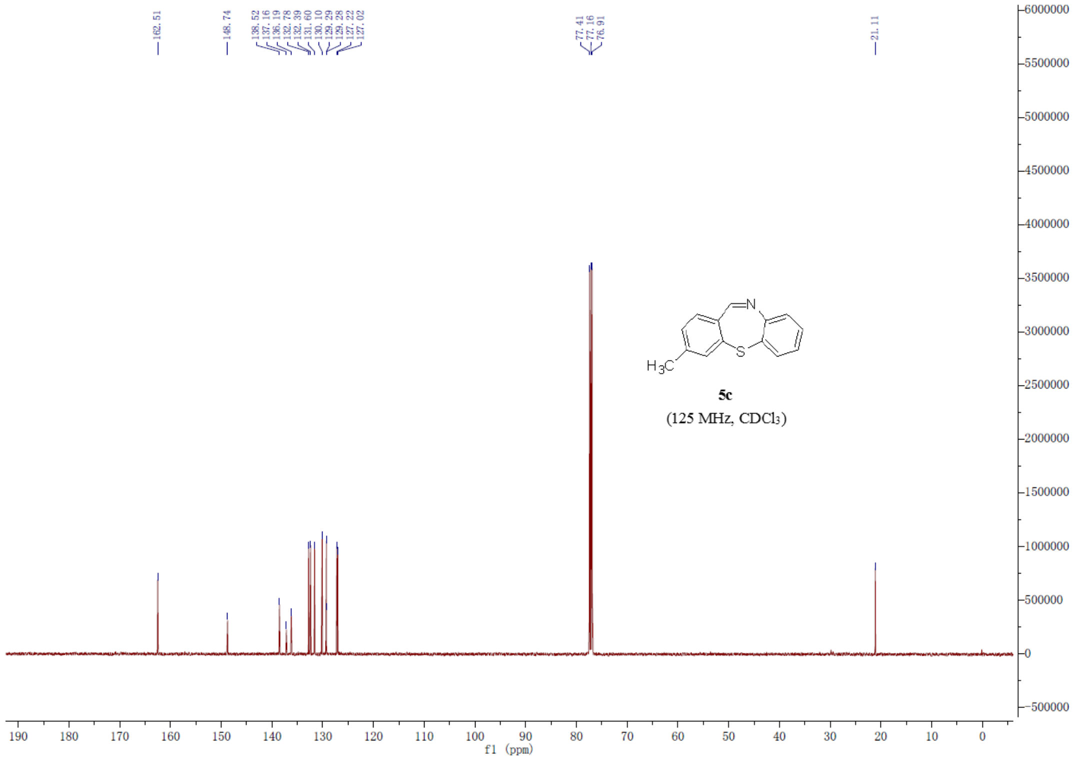

Figure S10: $^{13}$C NMR ($CDCl_3$) spectrum of Compound (**5c**)

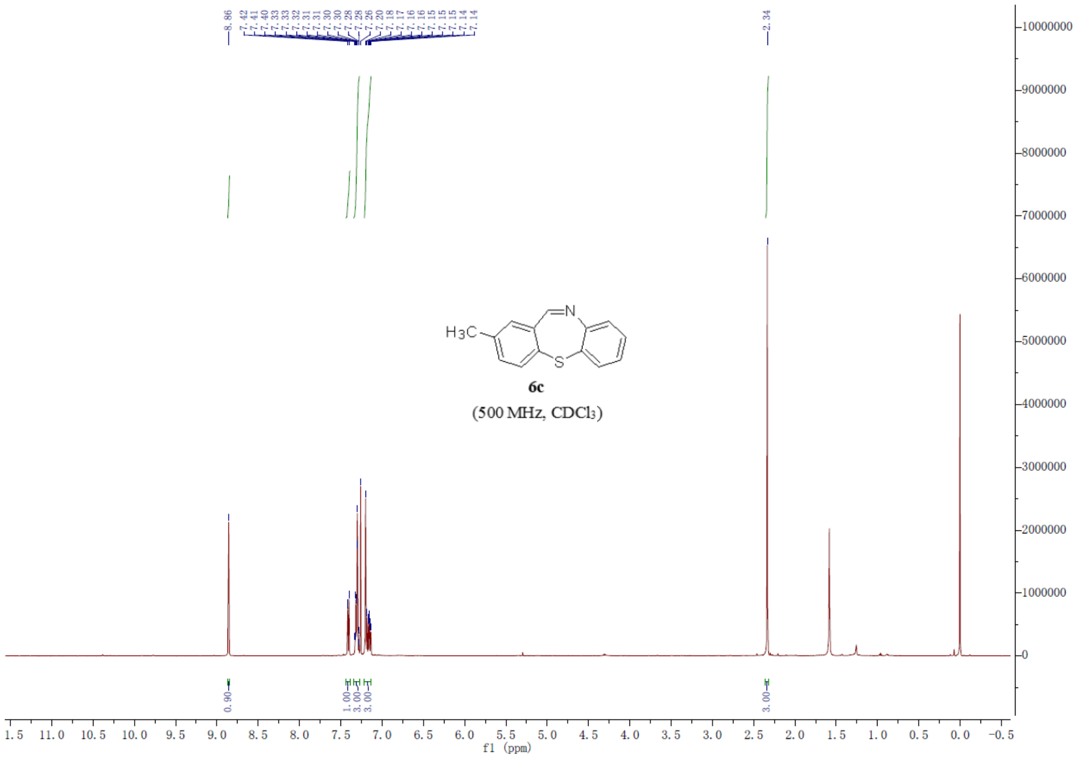


Figure S11: $^{1}$H NMR ($CDCl_3$) spectrum of Compound (**6c**)

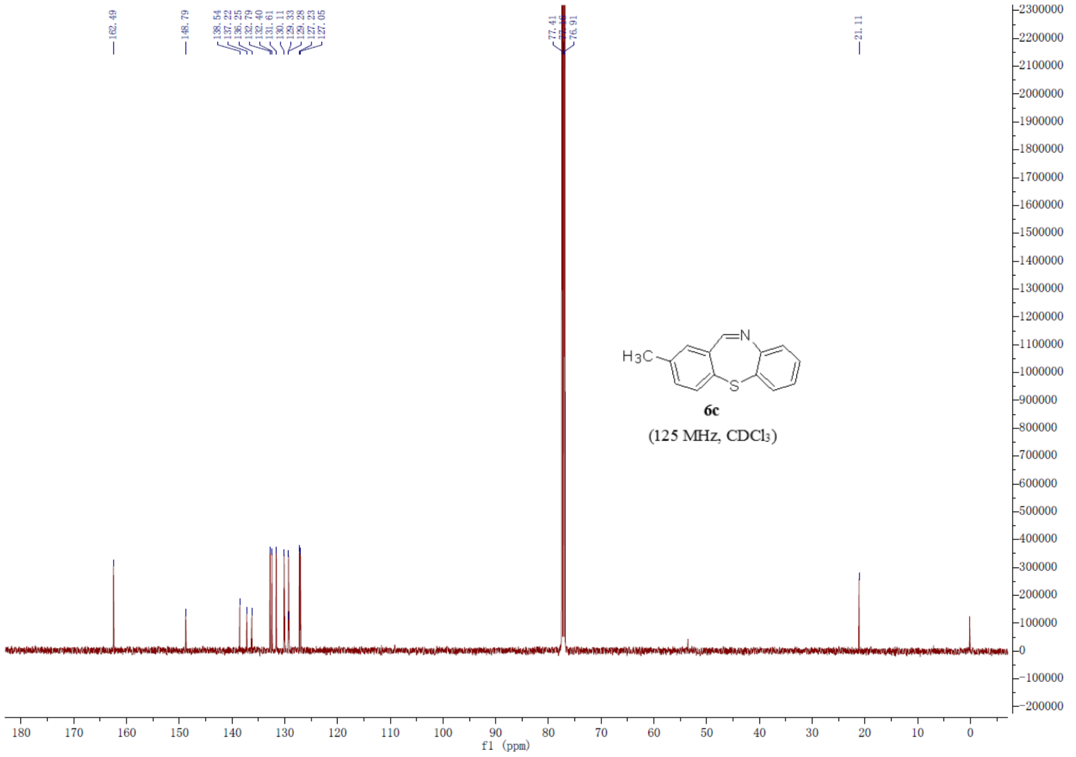


Figure S12: $^{13}$C NMR ($CDCl_3$) spectrum of Compound (**6c**)

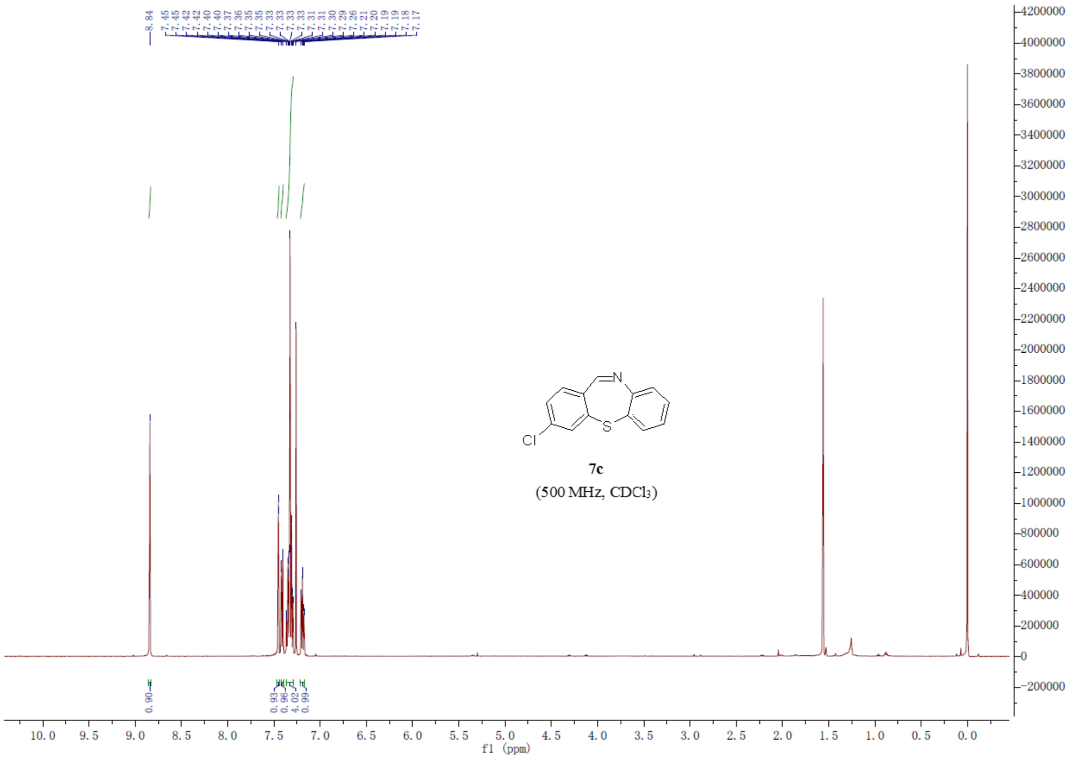


Figure S13: $^{1}$H NMR ($CDCl_3$) spectrum of Compound (**7c**)

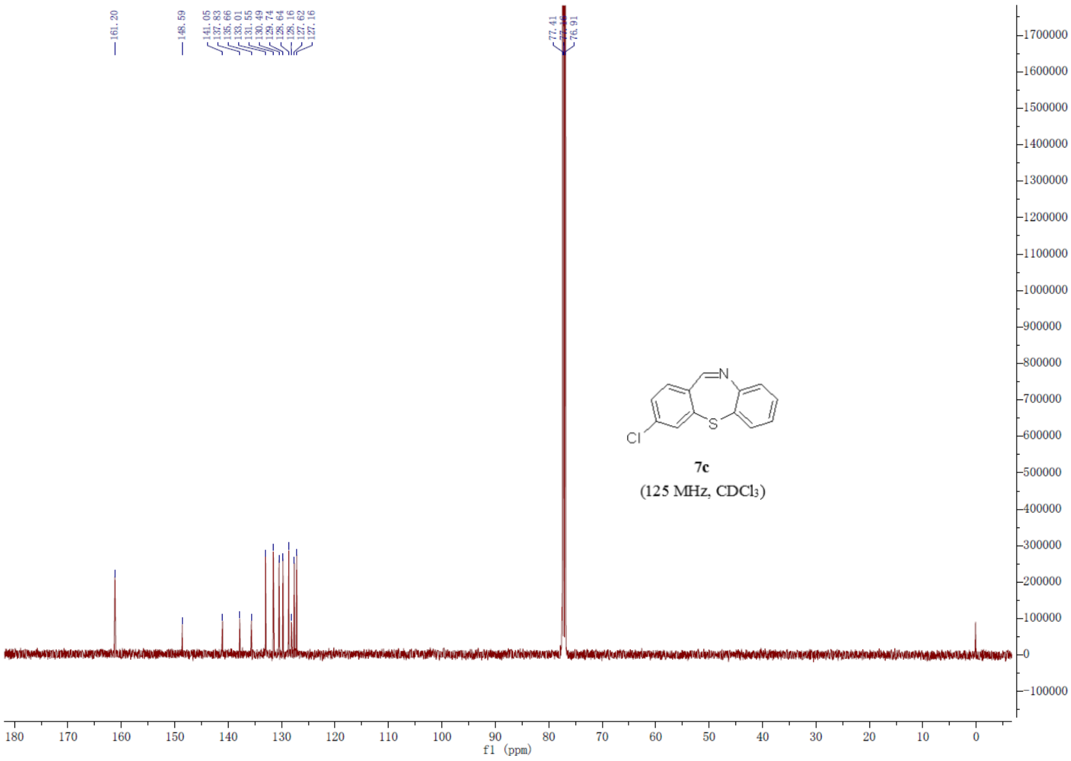


Figure S14: $^{13}$C NMR ($CDCl_3$) spectrum of Compound (**7c**)

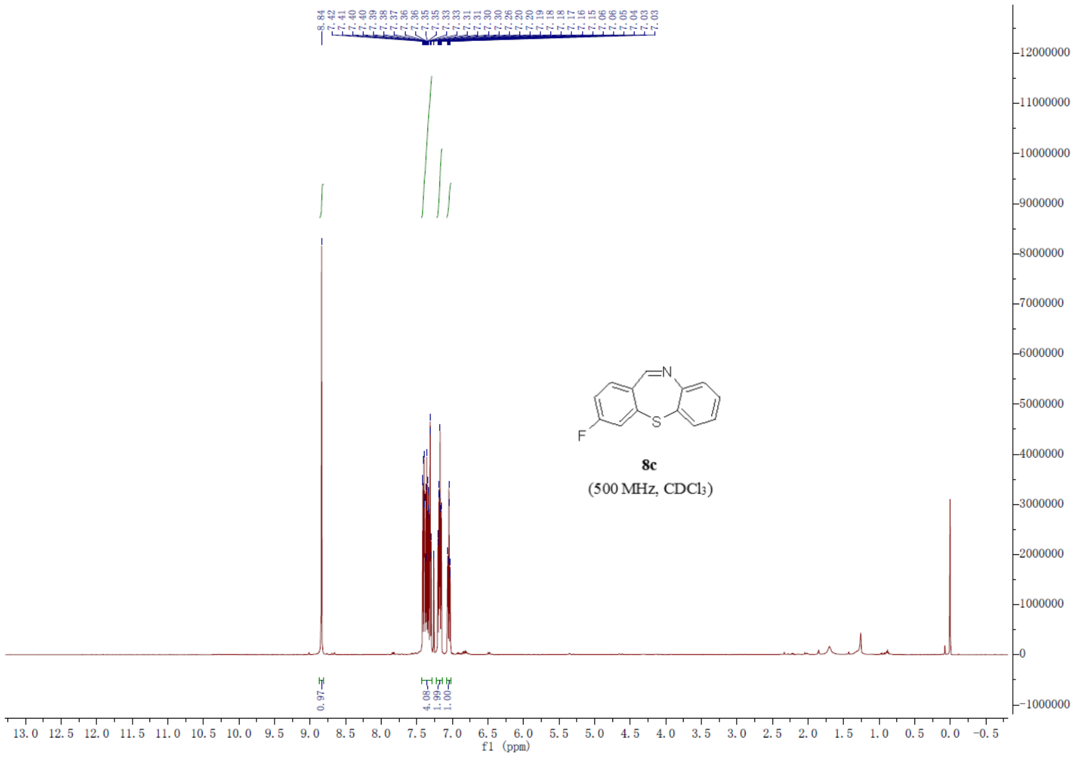


Figure S15: $^{1}$H NMR ($CDCl_3$) spectrum of Compound (**8c**)

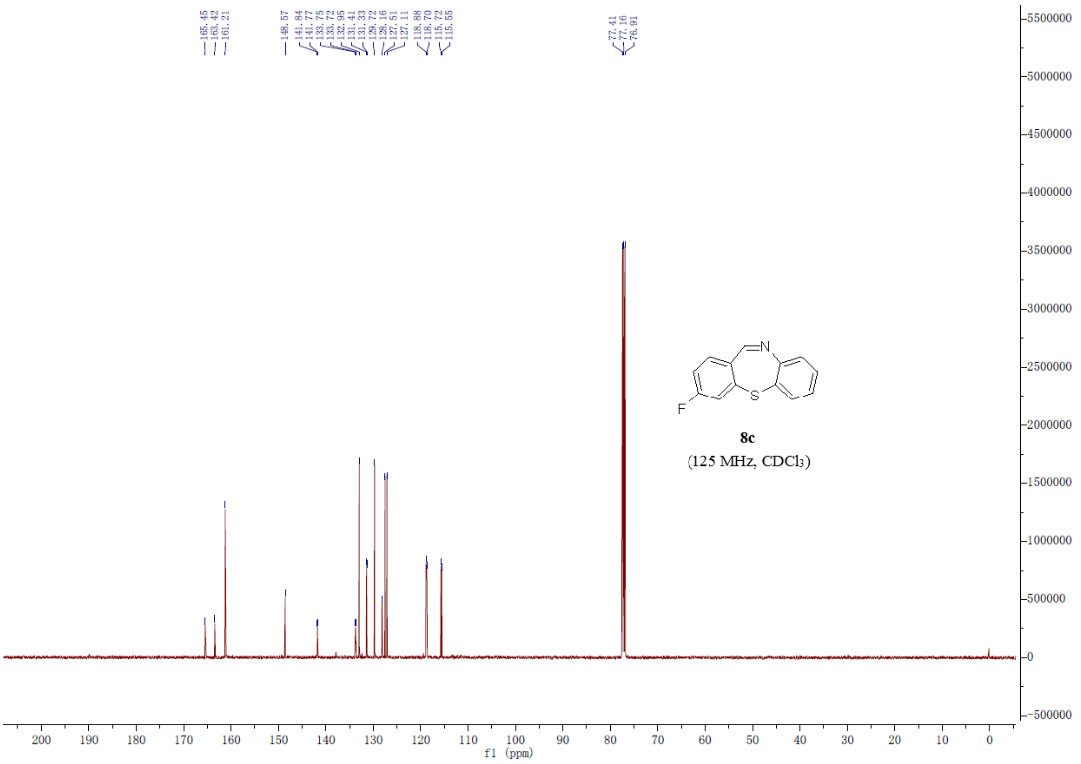


Figure S16: $^{13}$C NMR ($CDCl_3$) spectrum of Compound (**8c**)

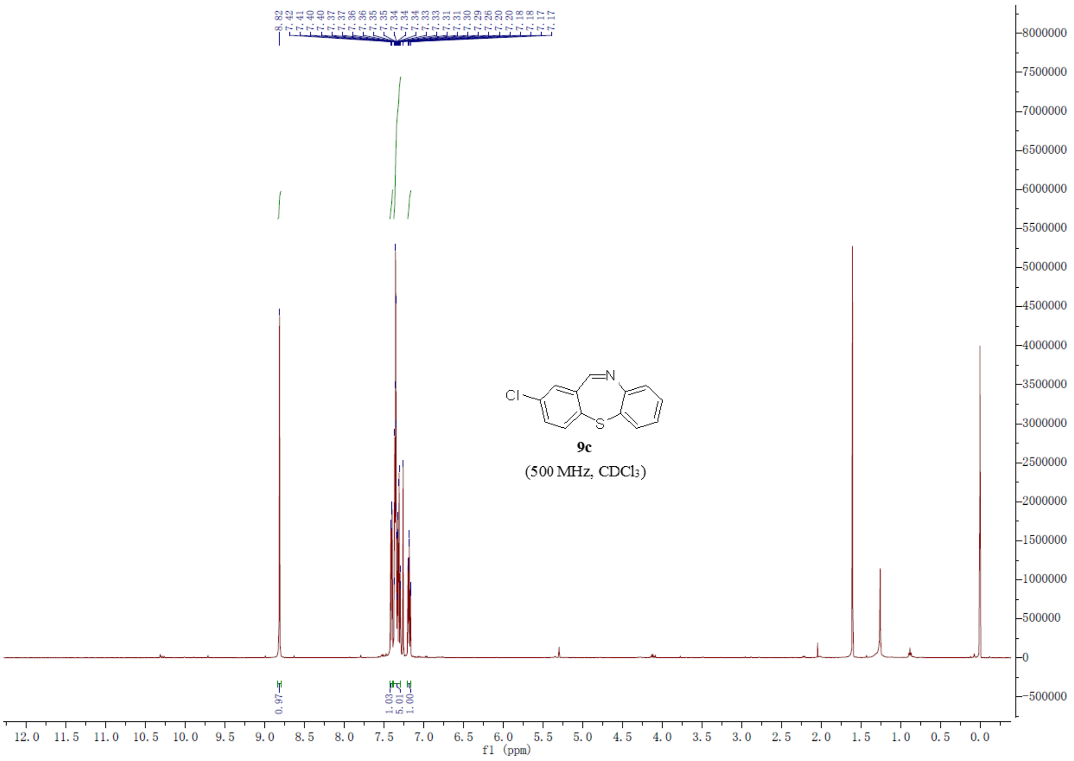


Figure S17: $^{1}$H NMR ($CDCl_3$) spectrum of Compound (**9c**)

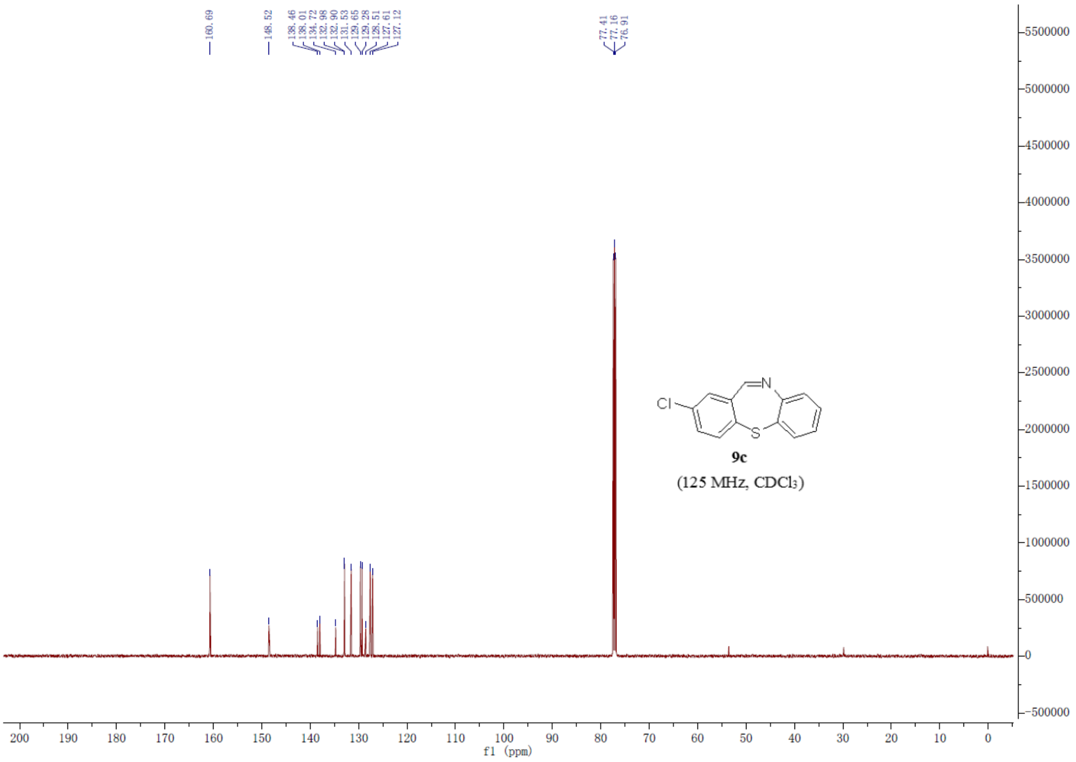


Figure S18: $^{13}$C NMR ($CDCl_3$) spectrum of Compound (**9c**)

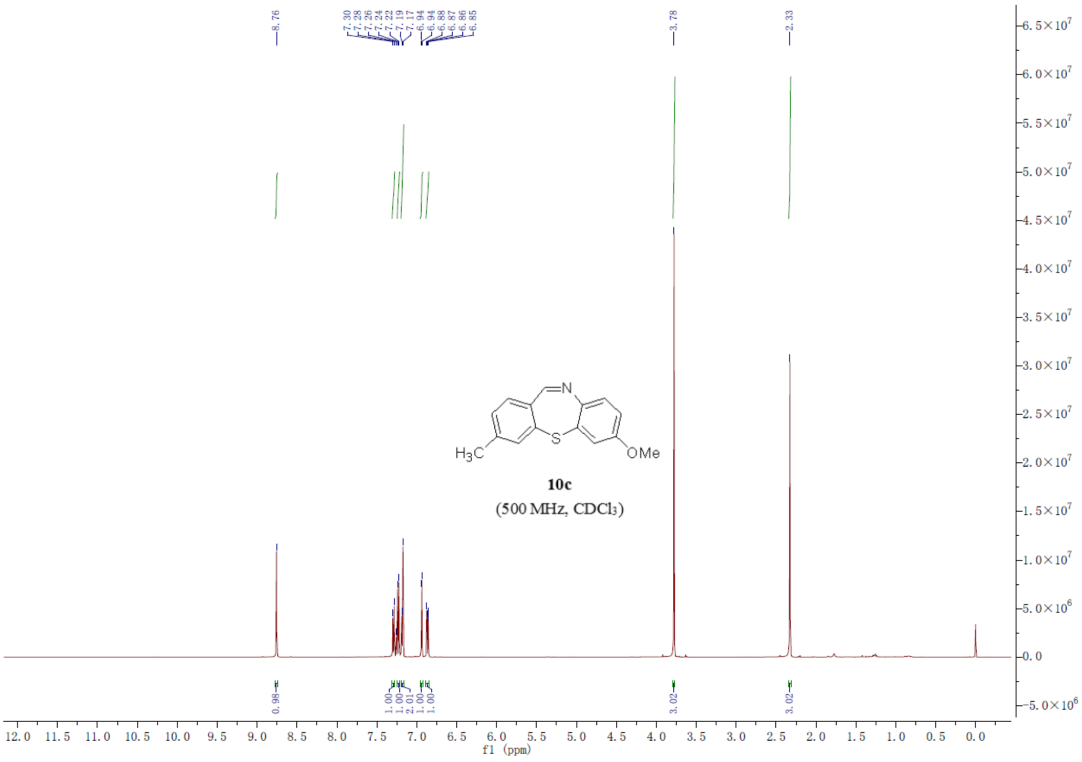


Figure S19: $^{1}$H NMR (CDCl$_3$) spectrum of Compound (**10c**)

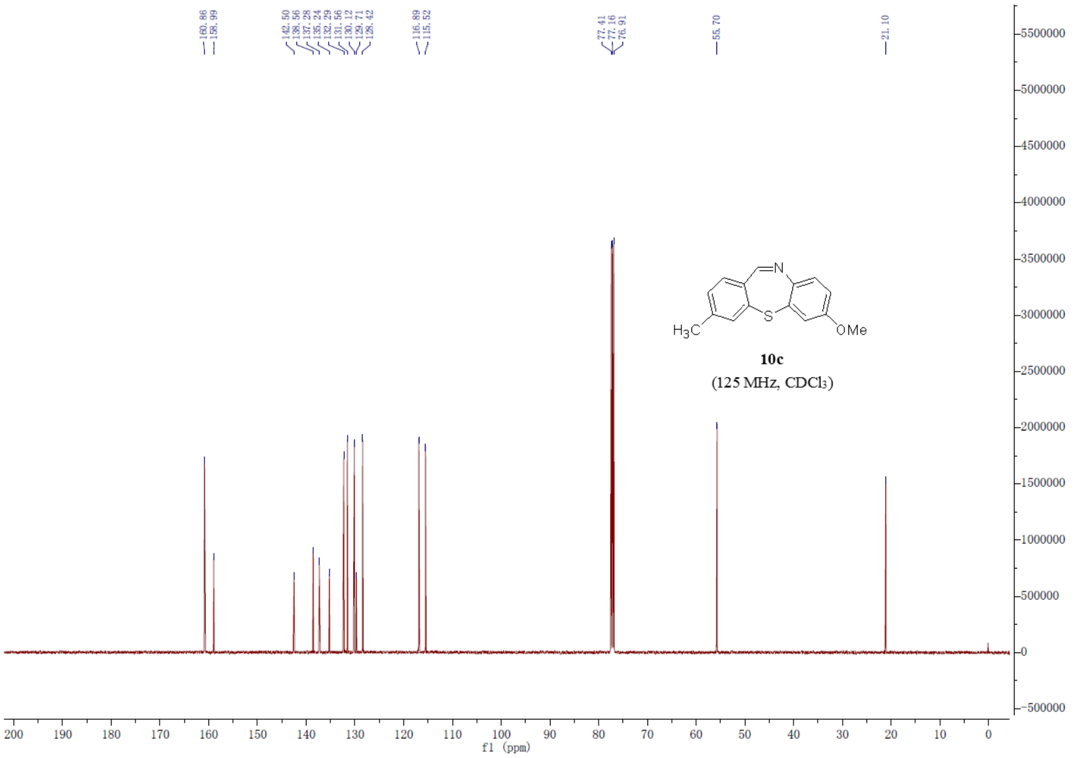


Figure S20: $^{13}$C NMR (CDCl$_3$) spectrum of Compound (**10c**)

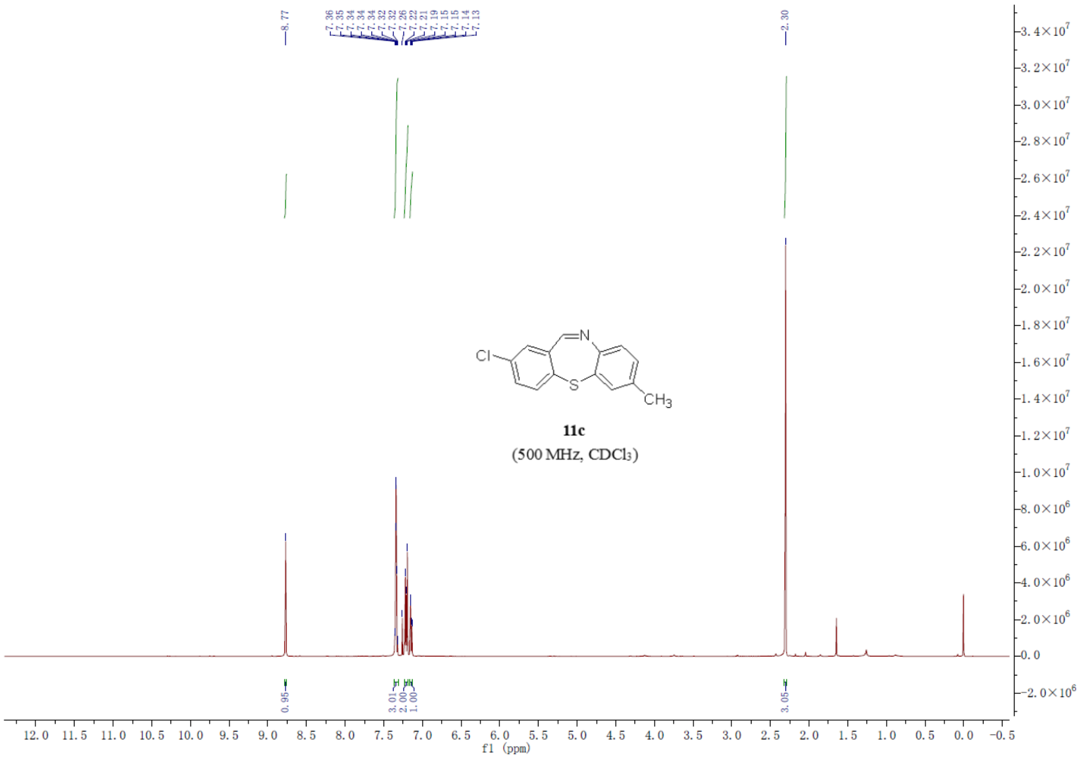

Figure S21: $^{1}$H NMR ($CDCl_3$) spectrum of Compound (**11c**)

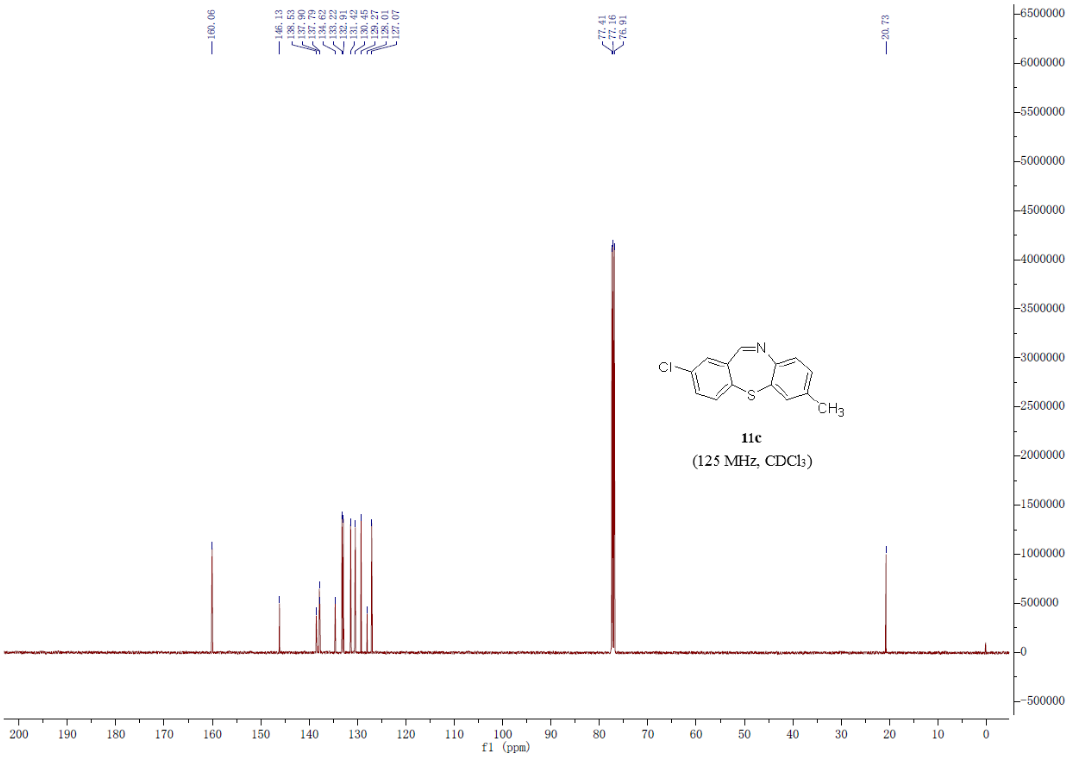

Figure S22: $^{13}$C NMR ($CDCl_3$) spectrum of Compound (**11c**)

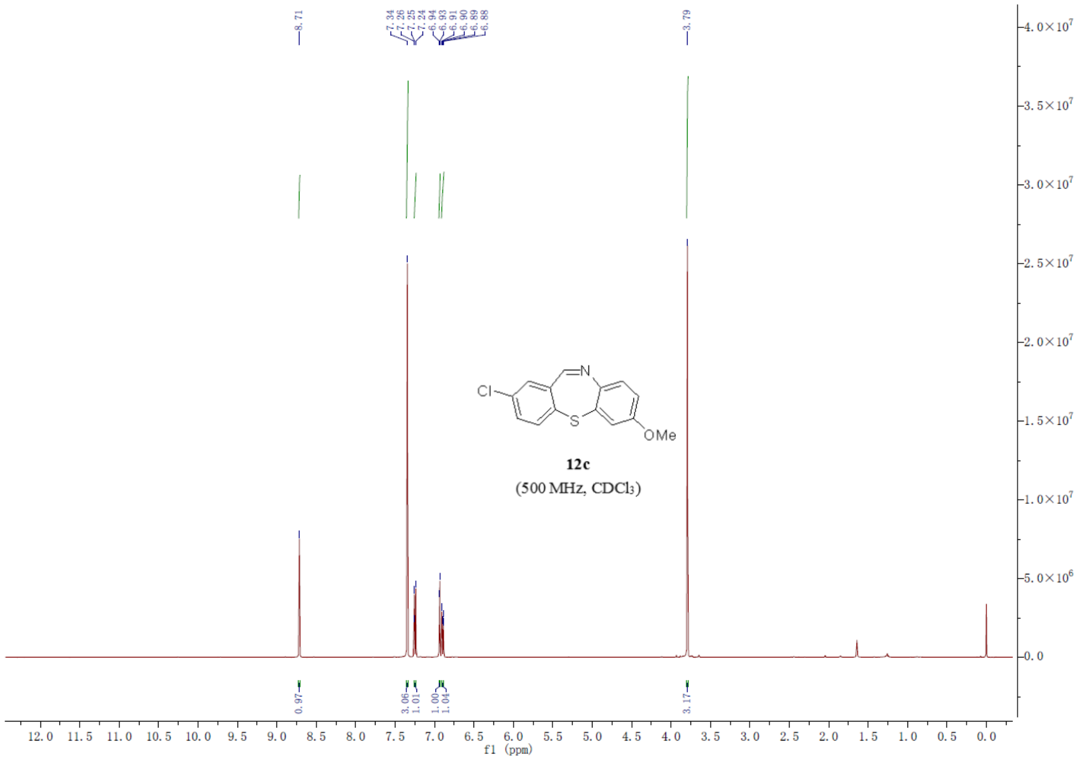


Figure S23: $^{1}$H NMR ($CDCl_3$) spectrum of Compound (**12c**)

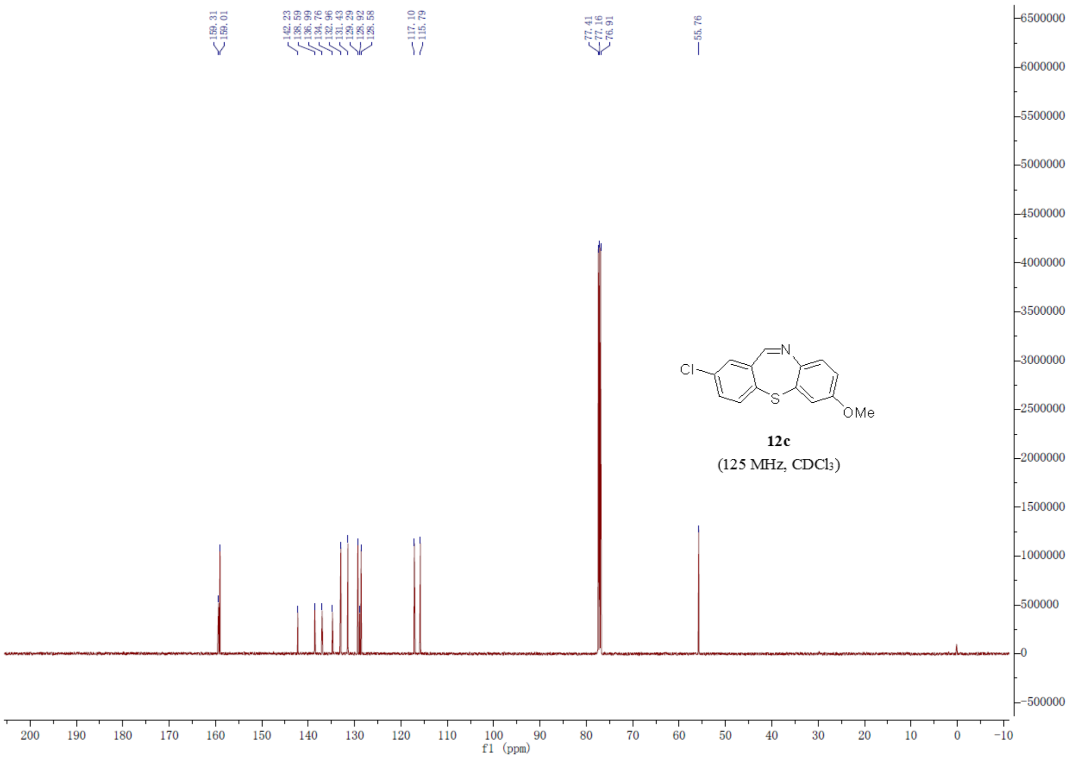


Figure S24: $^{13}$C NMR ($CDCl_3$) spectrum of Compound (**12c**)

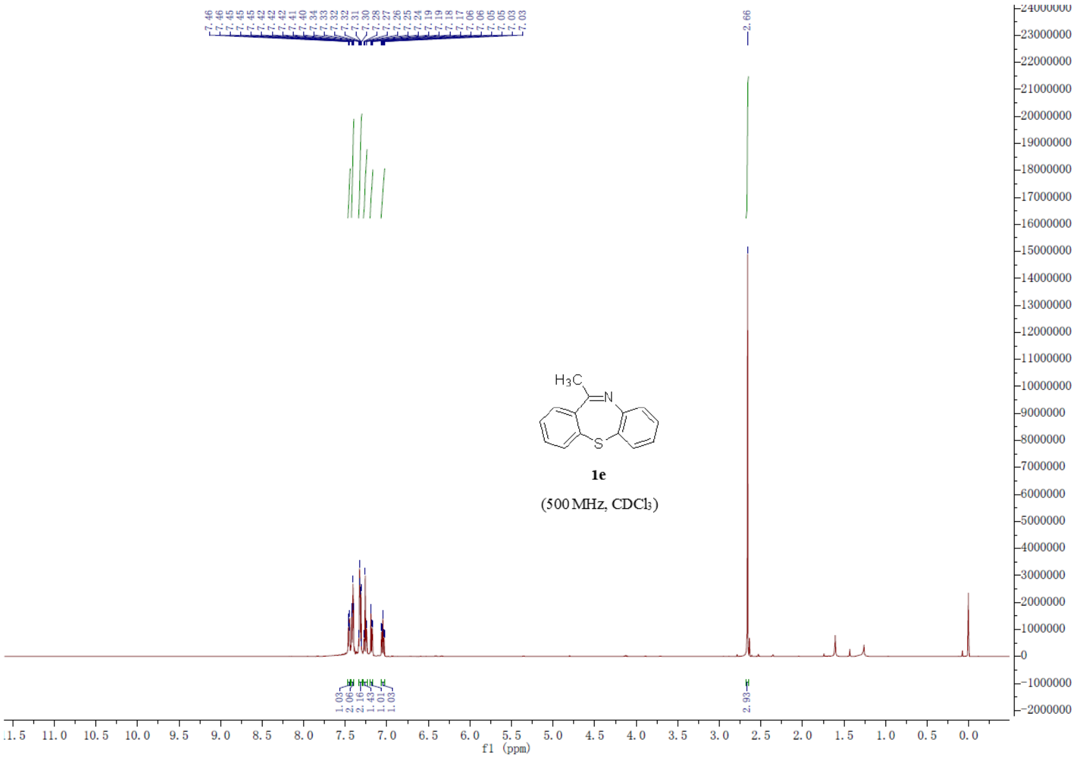


Figure S25: $^{1}$H NMR ($CDCl_3$) spectrum of Compound (**1e**)

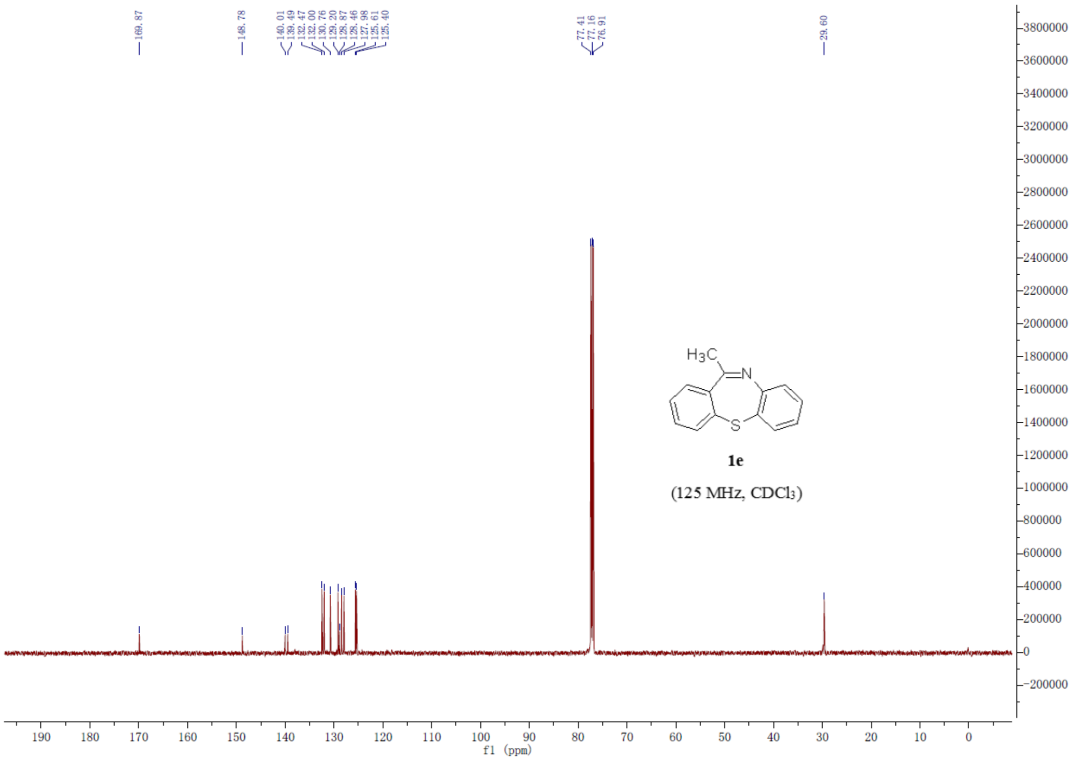


Figure S26: $^{13}$C NMR ($CDCl_3$) spectrum of Compound (**1e**)

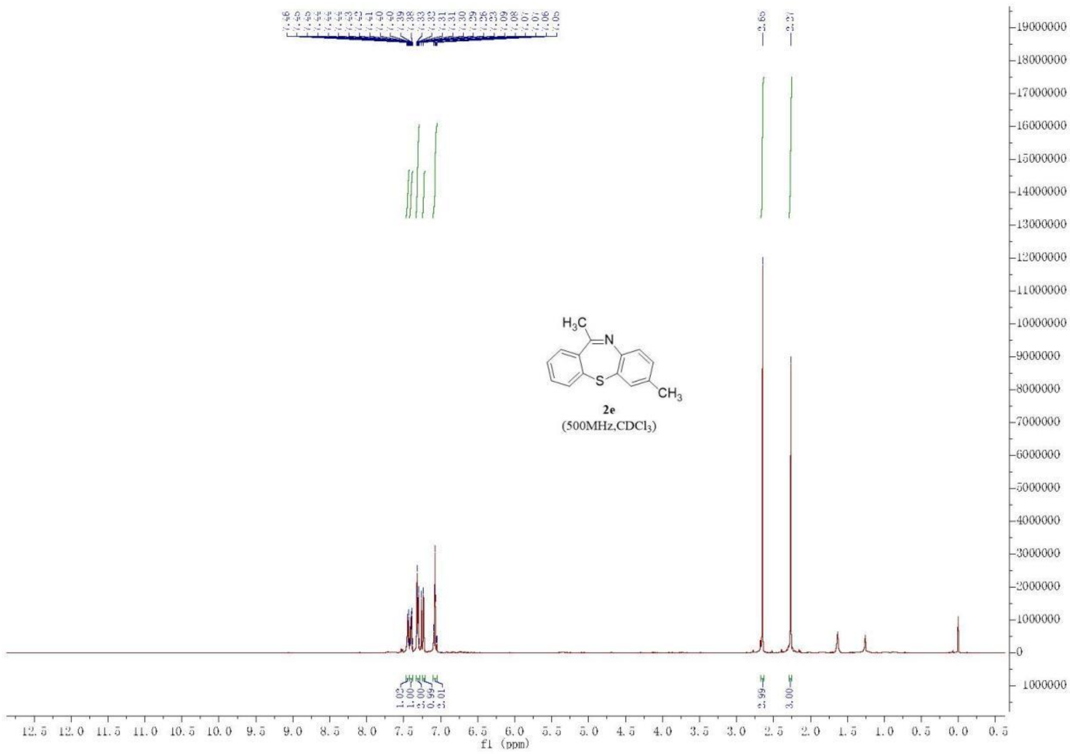


Figure S27: $^{1}$H NMR ($CDCl_3$) spectrum of Compound (**2e**)

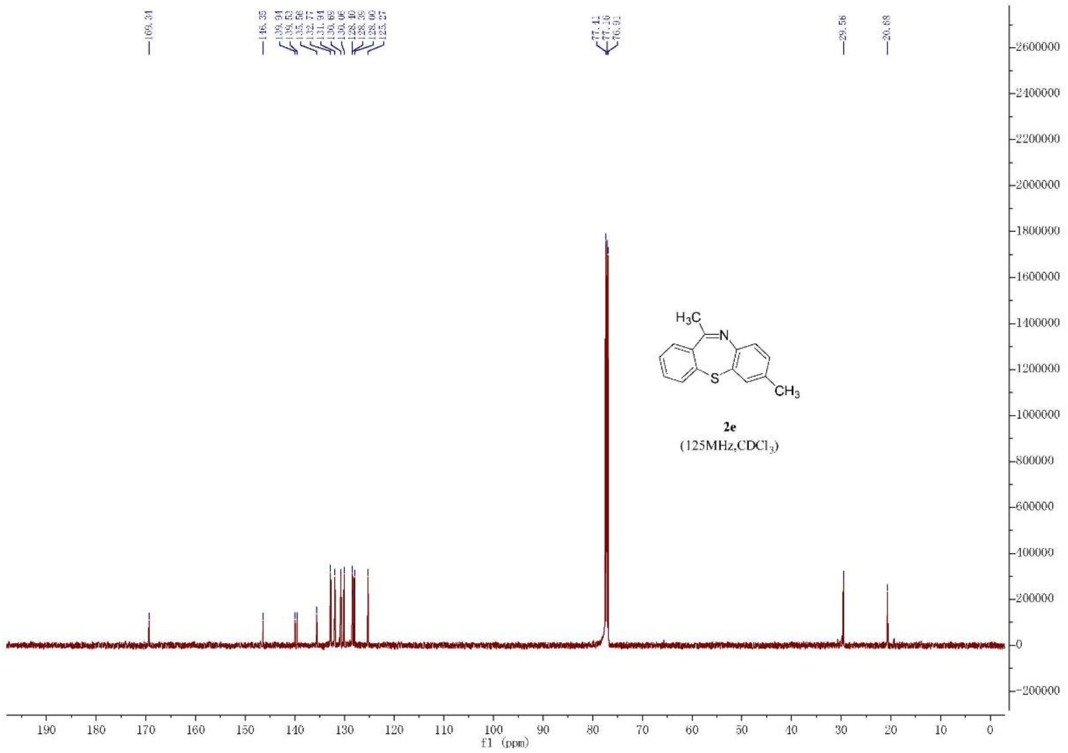


Figure S28: $^{13}$C NMR ($CDCl_3$) spectrum of Compound (**2e**)

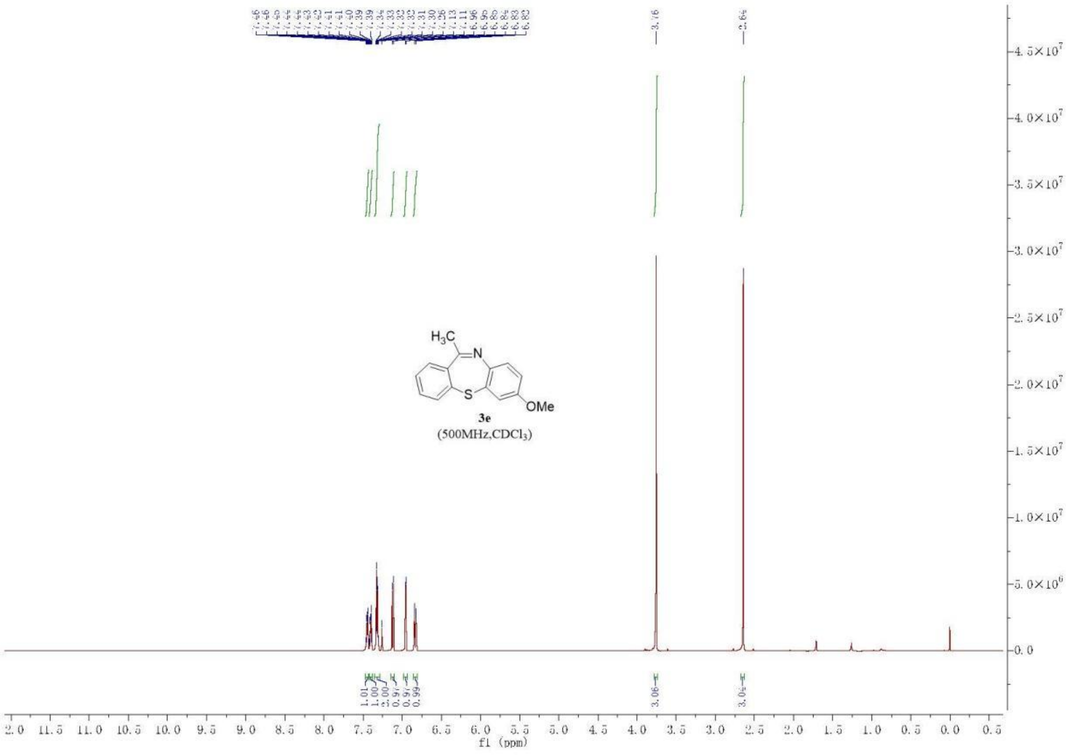

Figure S29: $^{1}$H NMR ($CDCl_3$) spectrum of Compound (**3e**)

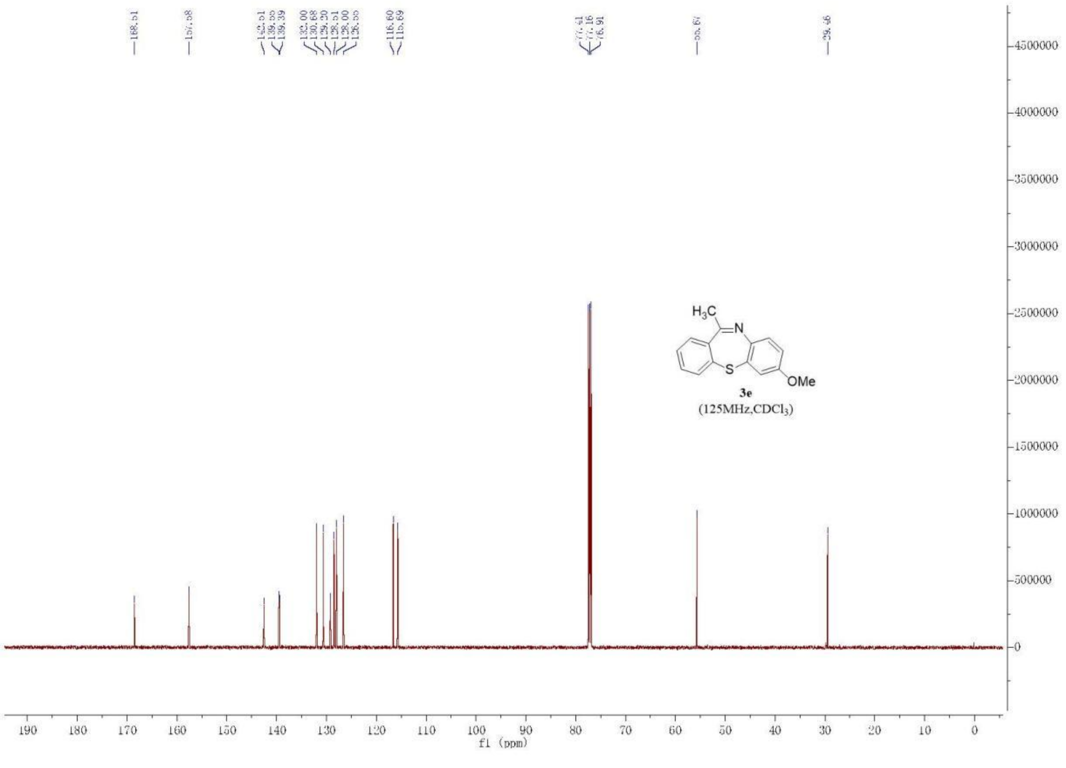

Figure S30: $^{13}$C NMR ($CDCl_3$) spectrum of Compound (**3e**)

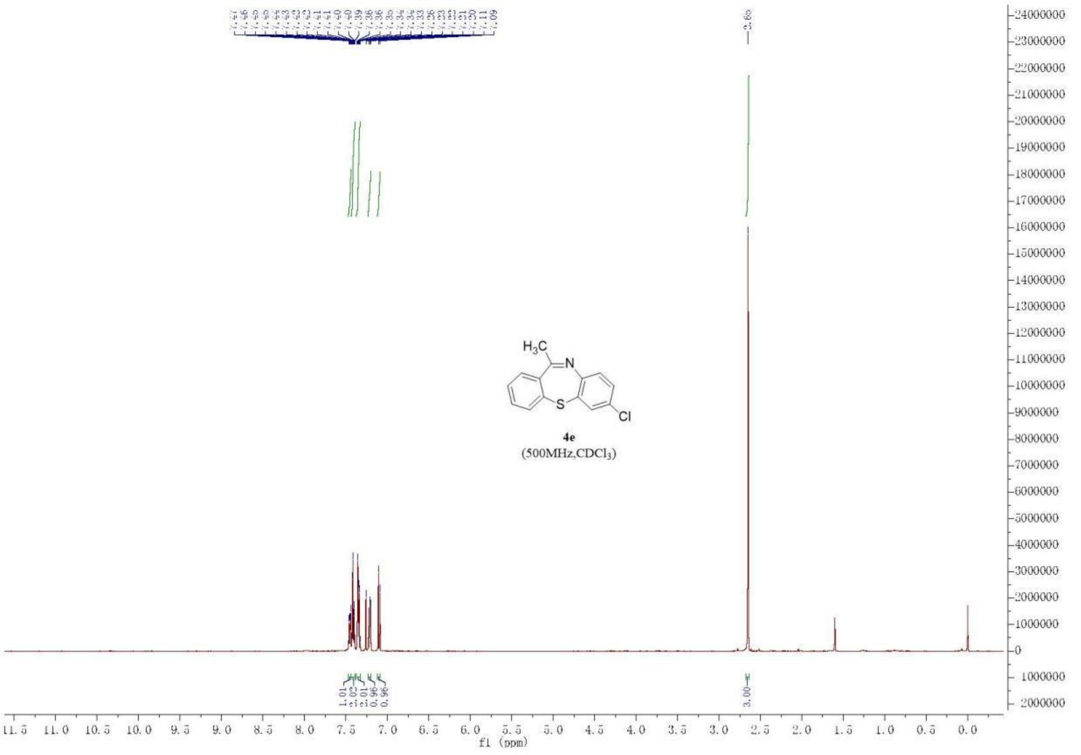


Figure S31: $^{1}$H NMR ($CDCl_3$) spectrum of Compound (**4e**)

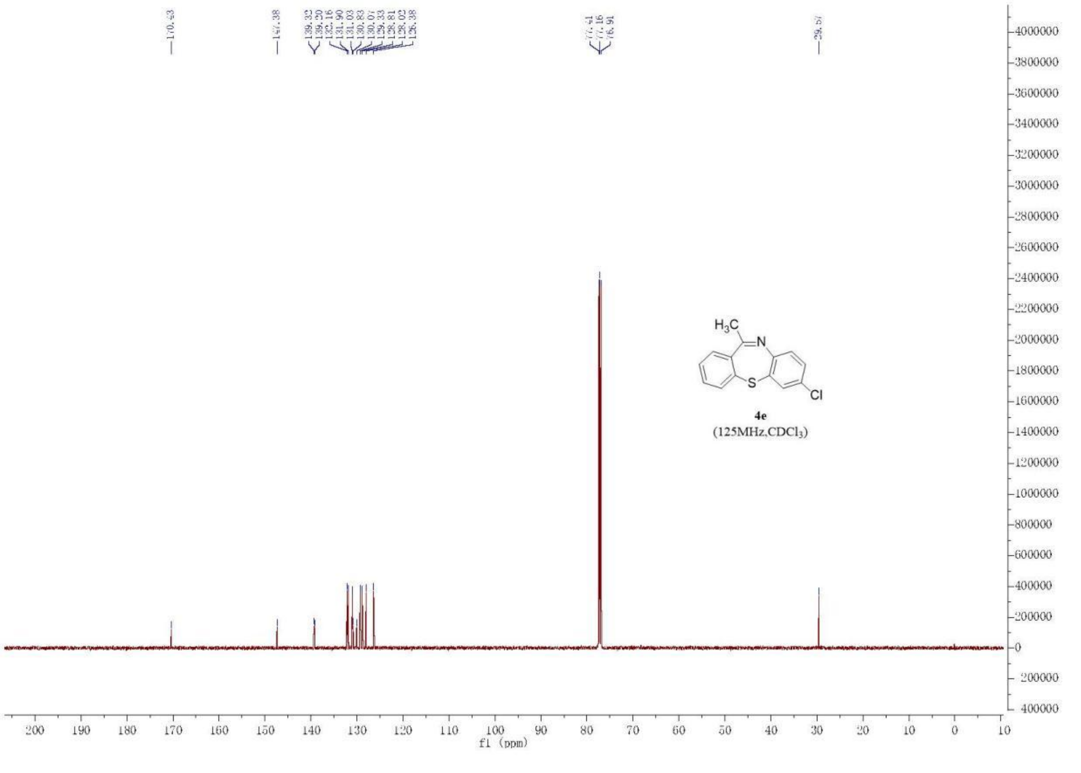


Figure S32: $^{13}$C NMR ($CDCl_3$) spectrum of Compound (**4e**)

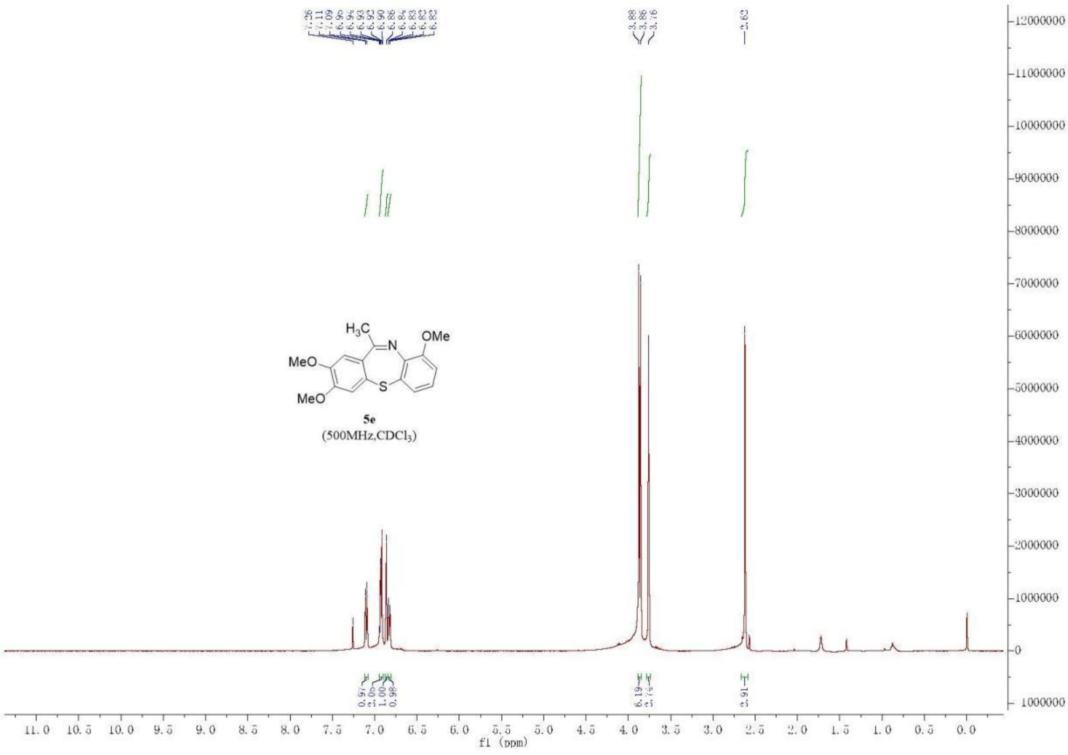

Figure S33: $^{1}H$ NMR ($CDCl_3$) spectrum of Compound (**5e**)

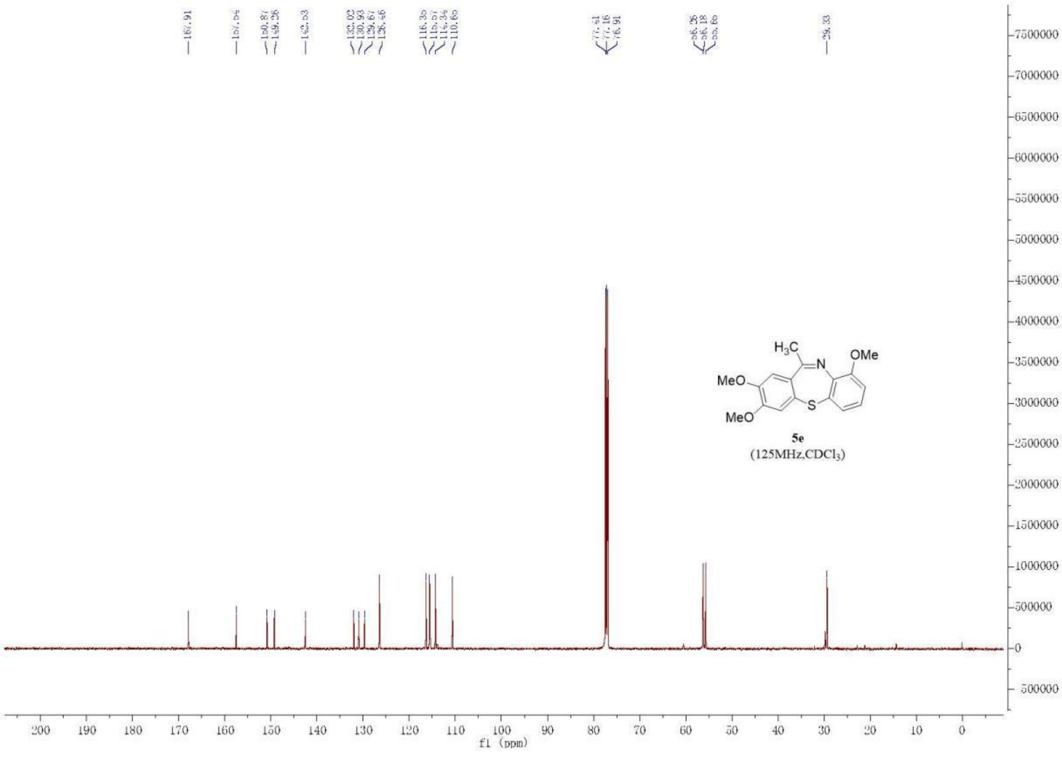

Figure S34: $^{13}C$ NMR ($CDCl_3$) spectrum of Compound (**5e**)
